# Evaluating the Economic Impact of the PedAMINES App in Reducing Medication Errors in Pediatric Emergency Care: Cost-Effectiveness Analysis

**DOI:** 10.2196/52077

**Published:** 2024-10-25

**Authors:** Loïc Brunner, Johan N Siebert, Frédéric Ehrler, Sergio Manzano, Joachim Marti

**Affiliations:** 1 Department of Epidemiology and Health Systems Center for Primary Care and Public Health (Unisanté) University of Lausanne Lausanne Switzerland; 2 Department of Pediatric Emergency Medicine Geneva Children's Hospital Geneva University Hospitals Geneva Switzerland; 3 Faculty of Medicine University of Geneva Geneva Switzerland; 4 Department of Radiology and Medical Informatics Division of Medical Information Sciences Geneva University Hospitals Geneva Switzerland

**Keywords:** adverse drug event, health information technology, pediatric care, emergency care, ambulance care, economic evaluation, cost-effectiveness, epinephrine, norepinephrine, midazolam, dopamine, evidence-based, medical app, medication error, pediatric, child, pediatric emergency care, PedAMINES, Pediatric Accurate Medication in Emergency Situations, Switzerland, child care, mobile phone

## Abstract

**Background:**

The administration of drugs in pediatric emergency care is a time-consuming process and is associated with a higher occurrence of medication errors compared with adult care. This is attributed to the intricacies of administration, which involve calculating doses based on the child’s weight or age. To mitigate the occurrence of adverse drug events (ADEs), the PedAMINES (Pediatric Accurate Medication in Emergency Situations; Geneva University Hospitals) mobile app has been developed. This app offers a step-by-step guide for preparing and administering pediatric drugs during emergency interventions by automating the dose calculation process. Although previous simulation-based randomized controlled trials conducted in emergency care have demonstrated the efficacy of the PedAMINES app in reducing drug administration errors, there is a lack of evidence regarding its economic implications.

**Objective:**

This study aims to evaluate the cost-effectiveness of implementing the PedAMINES app for 4 emergency drugs: epinephrine, norepinephrine, dopamine, and midazolam.

**Methods:**

The economic evaluation was conducted by combining hospital data from 2019, previous trial outcomes, information extracted from existing literature, and PedAMINES maintenance costs. The cost per avoided medication error was calculated, along with the number of administrations needed to achieve a positive return on investment. Subsequently, Monte Carlo simulations were used to identify the key parameters contributing to result uncertainty.

**Results:**

The study revealed the number of preventable errors per administration for the 4 examined drugs: 0.513 for epinephrine, 0.484 for norepinephrine, 0.500 for dopamine, and 0.671 for midazolam. The cost-effectiveness ratios per ADE prevented were computed as follows: US $4808 for epinephrine, US $9705 for norepinephrine, US $6957 for dopamine, and US $2074 for midazolam. Accounting for the economic impact of ADEs, the analysis estimated that 16 administrations of epinephrine, 17 of norepinephrine and dopamine, and 13 of midazolam would be required to attain a positive return on investment. This corresponds to roughly one-third of the annual administrations at a major university hospital in Switzerland. The primary factors influencing the uncertainty in the estimated cost per ADE include the cost of maintenance of the app, the likelihood of an ADE resulting from an administration error, and the frequency of underdosing in the trial’s control group.

**Conclusions:**

A dedicated mobile app presents an economically viable solution to alleviate the health and economic burden of drug administration errors in in-hospital pediatric emergency care. The widespread adoption of this app is advocated to pool costs and extend the benefits on a national scale in Switzerland.

## Introduction

Medication errors are among the most common medical errors, with a significant health and economic impact on health care systems [1]. Incorrect drug doses increase the risk of adverse drug events (ADEs), which can adversely affect patient health outcomes, consume human and material resources, and increase health care costs [2-5]. Pediatric patients are at increased risk because most drugs administered intravenously to children are provided in vials originally prepared for the adult population, which must be dosed and prepared according to each child’s individual weight, varying widely between age groups [6,7]. This risk may be further increased in pediatric emergency departments (PED) and pediatric intensive care units (PICUs) due to the complexity of stressful and high-risk situations encountered [8-10]. In addition, comorbidities and poor health status increase the likelihood of ADE [11,12]. Although health information technology (HIT) plays an increasingly important role in modern medicine, notably by improving the safety of the medication process [13-17], there is limited evidence of its effectiveness and efficiency [15], particularly in pediatrics [16-19]. Therefore, the need for clinical and cost-effectiveness evidence to support the implementation of digital health interventions to prevent medication error-related ADE in pediatric emergency care has become increasingly important.

Previous trials have demonstrated the ability of a mobile medical app, PedAMINES (Pediatric Accurate Medication in Emergency Situations; Geneva University Hospitals), to significantly reduce out-of-hospital and in-hospital medication errors for intravenous drug administration compared with conventional preparation methods during simulation-based pediatric resuscitation [20,21]. The app guides the user through the preparation and administration of drugs by automatically calculating the correct weight-based doses and providing a detailed preparation sequence. This study evaluated the cost-effectiveness and net economic benefit of using the app compared with conventional drug preparation methods to reduce the rates of medication errors and ADEs during intravenous drug administration in pediatric emergency care in a tertiary hospital. The hypothesis was that the use of the app could be a cost-effective strategy at the societal level to reduce medication errors and the associated economic burden in pediatric emergency medicine.

## Methods

### Intervention

PedAMINES provides automatic calculation support for the injection of drugs at pediatric doses. Registered as a medical device, it consists of 2 main parts. The first part features a welcome screen where the user is prompted to enter the patient’s weight or age. Once this information is entered, the user is directed to a screen where each of the drugs listed for direct intravenous injection or continuous infusion can be selected from a menu on the left-hand side, with a detailed preparation procedure displayed on the right-hand panel, following a standardized and simplified path. The second part is an interface for editing, managing, and sharing drug lists with other users. Additional information about the app’s interface has been published previously [17]. Multicenter randomized controlled trials have demonstrated the effectiveness of the app in reducing pediatric dosing errors during emergency drug injections in life-threatening critical situations [20,21]. Data from these trials are used below to examine the app’s cost-effectiveness.

### Economic Evaluation

Initially, the reduction in error rates for direct intravenous administration of pediatric doses of epinephrine using the app was evaluated in the prehospital setting [20]. Epinephrine was selected due to its frequent use as a first-line agent in emergency situations, especially in pediatric cardiopulmonary resuscitation (CPR) and its extensive coverage in scientific literature, making it a standard reference and comparator drug. Subsequently, the findings from prehospital settings were extrapolated to the PICU environment of a tertiary hospital in Switzerland.

In the economic evaluation, the costs (ie, maintenance, update, and training costs) as well as the health benefits (ie, avoided administration errors with associated consequences) and nonhealth benefits (avoided costs, time saved) of using the app in pediatric care at the Geneva University Hospitals were considered. First, a cost-effectiveness analysis was performed, expressed as the cost per error avoided. Second, a simple decision-analytic model was used to translate the reduction in error rates into prevented ADEs of varying severity and, ultimately, avoided costs. The cost per ADE avoided was then used to determine the minimum number of patients per year that would need to be treated with the app for the intervention to be cost-neutral.

The analysis was then extended to another commonly used direct intravenous drug in the prehospital setting, midazolam [20]. Finally, to assess the potential benefits of using the app in multiple contexts, the app’s use for the continuous infusion of norepinephrine and dopamine in the in-hospital emergency care setting (ie, PED) was further investigated. The choice of these 2 vasopressors was based on previous experience with their use [21]. In addition, dosing recommendations and preparation methods for the continuous infusion of norepinephrine were similar to those for epinephrine [22]. The costs and benefits of using the application were evaluated separately for each drug to provide a conservative perspective on implementing the application for only one drug at a time.

### Intervention Costs

We first estimated the direct costs of using the app from the provider’s perspective. The upfront investment cost of developing the app was not included in the cost-effectiveness calculations (ie, US $186,026), but we did include the annual recurring costs of maintenance and upgrades. These costs were estimated to be 20% of the development costs (ie, US $37,205) per year.

The direct costs of using the app from the provider’s perspective were initially estimated. The upfront investment cost of developing the app, totaling US $186,026, was not included in the cost-effectiveness calculations [23,24]. However, annual recurring costs for maintenance and upgrades, estimated at 20% of the development costs (US $37,205 per year), were included. It was assumed that hardware costs would be zero, as the app can be easily installed on standard hospital equipment such as smartphones or tablets commonly used in ambulances [25,26]. In addition, the cost of an initial 15-minute basic training session on the app, along with annual 15-minute refresher sessions for medical staff, was included. These training costs were valued based on the salaries of nurses and physicians in pediatric care at the institution’s accounting services, resulting in an annual training cost of approximately US $5 for nurses and US $7.5 for physicians. For instance, in 2019, the PED employed 60 full-time equivalent nurses and 22.8 full-time equivalent physicians, resulting in a total annual training cost of about US $1400.

### Intervention Outcomes and Benefits

The medication errors considered in the analysis are defined as deviations from the correct weight-based dose by more than or less than 10% [27]. The number of errors reported in patients with and without the assistance of the app for emergency drug preparation was previously identified [20]. Using this information, a ratio of preventable errors per epinephrine administration was calculated. Subsequently, the maintenance and training costs outlined earlier were used to quantify the resources invested per error avoided.

To estimate the avoided costs associated with the reduction in errors (*N_err_*), the potentially prevented adverse drug events (*N_ADE_*) attributable to error reduction were then estimated using scientific literature and hospital data as sources of information. ADEs were defined according to the criteria of the National Coordinating Council for Medication Error Reporting and Prevention, categorized as follows: (1) temporary patient harm requiring intervention (*Category E*) and (2) temporary patient harm requiring initial or prolonged hospitalization (*Category F*) [28]. The probability that a medication error would result in an ADE was calculated, along with the probability that an error would result in temporary harm requiring either intervention (*N_Cat E_*) or prolonged hospitalization (*N_Cat F_*) [29-32]. Due to the lack of evidence on mortality from medication errors in pediatric emergency care, a conservative assumption was made that an error would never result in patient death. Although some patients may experience multiple concurrent ADEs in 15% of cases [29], for simplicity, it was assumed that an error could result in at most 1 ADE.

NADE = N_err_ ⋅ P_(ADE)_

NCat E = NADE ⋅ P_(Cat E)_

NCat F = NADE ⋅ P_(Cat F)_

These ADEs were then converted into costs. For ADEs requiring intervention, the cost consequences were estimated as the average excess reimbursement received by the hospital for complication-related hospital stays (ie, +90%; Multimedia Appendix 1), which was applied to the average cost of a hospital stay. Studies reporting outcomes for ADEs requiring prolonged length of stay (LOS) in adult [33,34] and pediatric [35,36] populations were reviewed. However, the heterogeneity of these studies, which was highly dependent on the setting and the type of drug studied, did not allow an exact number of avoidable hospital days to be determined. Therefore, the increase in LOS due to opioid-related adverse events in surgical hospitalizations, equivalent to an additional 0.64 days [33], was used as a reference. The cost of these additional days resulting from an ADE was evaluated using the average daily hospital cost at the Geneva University Hospitals.

As an additional potential benefit, the economic value of time saved by using the app compared with standard drug dose calculations was included. The previously identified time savings per drug injection [20] were valued using corresponding wage information.

### Economic Analysis

All costs and outcomes were identified for a single year, 2019. The cost and outcome data described above were used to calculate incremental cost-effectiveness ratios (ICERs; *ICER_err_* and *ICER_ADE_*) [37]. The ICER is expressed as the ratio of the difference in cost between 2 strategies (ie, between the app [*C_app_*] and conventional [*C_conv_*] drug preparation methods) to the difference in effectiveness between the app (*E_app_*) and the conventional method (*E_conv_*).


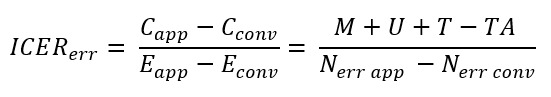



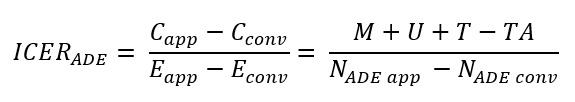


Subsequently, an analysis was conducted that included the economic benefit of avoiding ADEs, and the results were expressed in terms of the minimum number of administrations required to achieve a positive return on investment (ROI). To perform this calculation, the costs of using the app (maintenance [*M*], update [*U*], and training costs [*T*]) were compared with its monetary benefits derived from the time saved during administration (*TA*).

A probabilistic sensitivity analysis of the parameters was then performed to reflect the uncertainty in the decision problem, using either a uniform or normal distribution where available (Table 1). The relative impact of uncertainty in key parameters on the number of uses required to achieve a positive ROI was also determined. In addition, Monte Carlo (MC) simulation [36] was used, involving random sampling based on probability distributions of the parameters, to provide further insights into the uncertainty surrounding the calculated values [38].

To maintain consistency with the timing of other data used and to mitigate the potential impacts of the COVID-19 pandemic on costs in 2020 and 2021, all costs in the following analyses were converted to US dollars based on the costs in CHF from the in-hospital trial conducted in 2019 [21] (exchange rate of CHF 1=US $1.03 as of December 31, 2019). All parameters used in the calculations are detailed for epinephrine (Table 1) and for norepinephrine, dopamine, and midazolam (Multimedia Appendix 2). Analyses were performed using Microsoft Excel 2016 (version 16.0.5254.1000).

**Table 1:** Variables and distributions used in the analysis.

Parameter	Distribution^a^	Mean	SD	CI 95%	Reference
Total number of epinephrine administrations investigated in the 2021 prehospital trial	Fixed value	76	—^b^	—	[20]
Preparation time to intravenous epinephrine administration in the experimental group (ie, with app support), in seconds	Normal	191.6	80.3	34.2-349	[20]
Time to intravenous epinephrine administration in the control group (ie, without app support), in seconds	Normal	201.1	73.4	56.2-344	[20]
Number of epinephrine overdoses (>10% of the prescribed dose) in the experimental group	Uniform	2.0	—	1.6-2.4	[20]
Number of epinephrine overdoses (>10% of prescribed dose) in the control group	Uniform	7.0	—	5.6-8.4	[20]
Number of epinephrine underdoses (>10% of prescribed dose) in the experimental group	Uniform	2.0	—	1.6-2.4	[20]
Number of epinephrine underdoses (>10% of prescribed dose) in the control group	Uniform	36.0	—	28.8-43.2	[20]
Probability of an ADE^c^	Normal	11.1%	1.1%	9.1%-13.5%	[28]
Probability of temporary patient harm and need for initial or prolonged hospitalization (category F)	Normal	3%	1.89%	0.6%-8%	[28]
Probability of temporary patient harm and complication (category E)	Normal	97%	1.89%	92%-99.4%	[28]
Daily cost of an inpatient stay in the PICU^d^, in US $, 2019	Uniform	229.7	—	183.8-275.7	Hospital accounting
Increased cost of hospitalization due to complications: 91.84% of PICU cost, in US $, 2019	Uniform	22,080.0	—	17,664-26,496	(Multimedia Appendix 1)
Costs of using and maintaining the app, including the software and resources used to use the app each year.	Uniform	37,205.3	—	29,764.2-44,646.3	Expert opinion + [23,24]
Cost of training a nurse in the PED^e^, in US $, 2019	Uniform	885.5	—	708.4-1052.6	Hospital accounting + expert opinion
Cost of training a physician in the PED, in US $, 2019	Uniform	522.7	—	418.1-627.3	Hospital accounting + expert opinion
Conditional length of stay, ie, the number of days of prolonged hospitalization in the event of an ADE.	Normal	0.6	0.1	0.4-0.9	[33]
US $: CHF exchange rate on 31 December 2019	Fixed value	1.03	—	—	[39]

^a^Where the information on the distribution of the variable was not available, a uniform distribution with a CI of ±20% was assumed.

^b^Not applicable.

^c^ADE: adverse drug event.

^d^PICU: pediatric intensive care unit.

^e^PED: pediatric emergency department.

### Dopamine, Norepinephrine, and Midazolam Replication

The app supports the preparation of multiple drugs for direct intravenous injection or continuous infusion. Therefore, the same methodology described above was applied to 3 other injectable drugs: norepinephrine, dopamine, and midazolam. The use of the app with dopamine and norepinephrine has been studied in the in-hospital setting [21], while its use with midazolam has been investigated in the same prehospital study as epinephrine [20], demonstrating the app’s value in different emergency contexts and preparation methods. All parameters for these additional analyses are provided in Multimedia Appendix 2. Replicating the analysis for different settings and types of drugs is crucial for reinforcing the external validity of the results. The selected drugs include 2 for direct intravenous administration in a prehospital setting by paramedics and 2 for continuous infusion in an in-hospital setting by nurses and physicians.

### Ethical Considerations

This study uses preexisting secondary data that was published before our analysis. The 2 principal trials referenced in this study underwent ethical review by the Geneva Cantonal Ethics Committee, as documented in the original publications [20,21]. For the first trial in 2016, the Geneva Cantonal Ethics Committee did not assign an approval code at the time, while for the second trial, the approval code was Req-2019-00773. As a result, additional ethical review was not required for this research. Furthermore, no individual participants from any of the studies used in this analysis can be identified.

## Results

### Cost Per Error Prevented Using the App for Epinephrine, Dopamine, Norepinephrine, and Midazolam

The number of errors and ADEs prevented using the app for direct intravenous epinephrine was calculated (Table 2). An error rate of 44 (57.9%) out of 76 injections was observed for epinephrine injection in the prehospital setting using conventional preparation methods, compared with 4 (5.4%) out of 74 injections using the app, resulting in a reduction in error rate of 53.8% (95% CI 38.4-66.4) [20]. The cost per prevented ADE was estimated at US $4808. Unfortunately, there was no available information on the cost of ADEs in Switzerland to contextualize this value. However, in Japan, the cost of an ADE was estimated at US $8258.23 [40], which is twice as high as the cost of preventing an ADE with the PedAMINES app. The literature provides limited information on the economic burden of ADEs in pediatric care. In the German emergency population, the mean cost of an ADE was €2743 (~US $2970) [41]. In the US population older than 64 years, the cost of ADE-related hospital admission has been estimated at US $17,796 [42]. These estimates highlight the challenges in determining ADE-related costs, which are highly dependent on context, the specific types of costs considered, and the population studied. However, they confirm the general range of costs associated with an ADE.

The number of errors ADEs prevented using the app for direct intravenous midazolam and continuous infusions of norepinephrine and dopamine was calculated (Table 2). Using the same calculation method as for epinephrine, the cost per error prevented for these 3 drugs was determined. It was found that preventing an ADE associated with norepinephrine would be the most expensive, while preventing an ADE associated with midazolam would be the least expensive. This analysis was repeated, accounting for an annual device cost of US $600 (acquisition of 2 tablets costing US $300 each annually), and showed similar results (more details in Multimedia Appendix 3).

**Table 2:** Preventable errors, preventable ADE^a^, cost per prevented error, and cost per prevented ADE using the app for epinephrine, dopamine, norepinephrine, and midazolam.

Drugs	Preventable errors per administration, n	Preventable ADE per administration, n	Cost per prevented error, in US $	Cost per prevented ADE, in US $
Epinephrine (direct IV^b^)	0.513	0.057	534	4808
Dopamine (continuous infusion)	0.500	0.056	772	6957
Norepinephrine (continuous infusion)	0.484	0.054	1077	9705
Midazolam (direct IV)	0.671	0.074	230	2074

^a^ADE: adverse drug event.

^b^IV: intravenous.

### Number of Drug Administrations Required to Achieve a Positive ROI Using the App for Epinephrine, Dopamine, Norepinephrine, and Midazolam

Overall, the number of epinephrine administrations required to achieve a positive ROI was found to be 16 (Table 3). Extrapolating to 2019, when the PICU of Geneva University Hospitals reported 40 administrations, a positive ROI could have been achieved for the remaining 24 administrations.

Similarly, a comparable number of drug administrations required to achieve a positive ROI with the app was estimated (Table 3). In 2019, using the app in our PICU with any of these drugs would have achieved a positive ROI.

The number of administrations required to achieve a positive ROI and the results of 10,000 Monte Carlo simulations for each of these drugs are shown in Table 3. In 95% of the simulations, a positive ROI would be obtained with the app after a maximum of 23 administrations of epinephrine, a maximum of 24 administrations of dopamine, a maximum of 35 administrations of norepinephrine, and a maximum of 19 administrations of midazolam.

**Table 3:** Number of drug administrations required to achieve a positive ROI^a^ and the result of Monte Carlo simulations.

Drugs	Drug administrations required to achieve a positive ROI (calculated), n	Drug administrations to achieve a positive ROI in 95% of cases (calculated), n	Total administrations at the PICU^b^ in 2019, n
Epinephrine (direct IV^c^)	16 (15.8)	23 (23.0)	40
Dopamine (continuous infusion)	17 (16.2)	24 (23.4)	100
Norepinephrine (continuous infusion)	17 (16.7)	35 (24.7)	141
Midazolam (direct IV)	13 (12.1)	19 (18.1)	250

^a^ROI: return on investment.

^b^PICU: pediatric intensive care unit.

^c^IV: intravenous.

### Sensitivity Analysis

**Figure 1:**
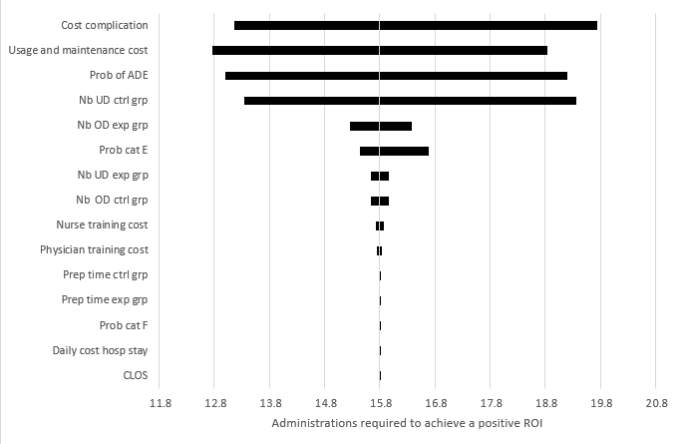
Deterministic sensitivity analysis (tornado plot): epinephrine. Relative importance of the uncertainty according to the variables used in the analysis. ADE: adverse drug event; cat: category; CLOS: conditional length of stay; ctrl grp: control group; epi: epinephrine; exp grp: experimental group; hosp: hospital; Nb: number; OD: overdoses; prob: probability; ROI: return on investment; UD: underdoses.

Sensitivity analysis of individual variables provided information on the relative importance of each parameter in the model used to predict the number of epinephrine administrations required to achieve a positive ROI. The independent effect of each parameter on the results is illustrated in a tornado plot (Figure 1). Maintenance and usage costs, the probability of occurrence of an ADE, and the frequency of underdosing in the control group were identified as the parameters with the strongest individual influence on the number of administrations required to achieve a positive ROI.

**Figure 2:**
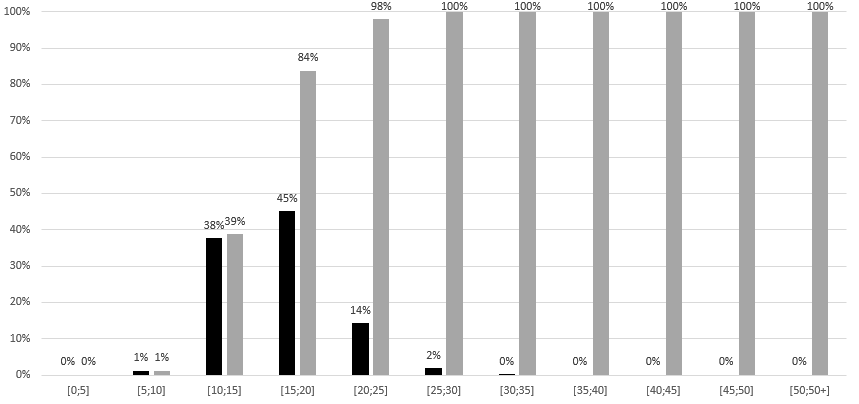
Proportion of simulations with a positive return on investment by the number of administrations: epinephrine. The number of administrations required to achieve a positive return on investment on 10,000 Monte Carlo simulations.

**Figure 3:**
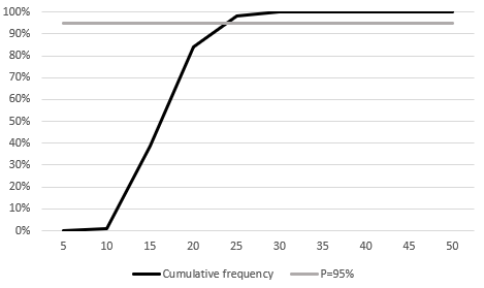
Sensitivity analysis, cumulative frequencies: epinephrine. Cumulative frequency of the number of administrations required to achieve a positive return on investment on 10,000 Monte Carlo simulations.

Probability distributions were used (Table 1) to assess the sensitivity of the results to parameter uncertainty. The distribution of results from the MC simulation is shown in a histogram (Figure 2). In this histogram, the gray bars represent the cumulative frequency, while the dark bars show the frequency of individual outcomes from the simulation. Subsequently, cumulative frequencies of these simulations based on probabilistic variations of the variables are displayed in Figure 3. In this figure, the grey line marks the threshold below which 95% of the simulations fall, while the black line represents the cumulative frequency of the Monte Carlo simulations. Out of 10,000 MC simulations, 95% achieved a positive ROI within 23 administrations. The sensitivity analysis reveals similar results for norepinephrine, midazolam, and dopamine. Therefore, cumulative simulation frequencies and tornado plots are provided in the supplementary material (Multimedia Appendices 4-5).

## Discussion

### Principal Findings

To the best of current knowledge, this study offers the most comprehensive economic analysis to date of using a medical mobile app to reduce medication errors and prevent ADEs in pediatric emergency care, compared with conventional drug preparation methods (Multimedia Appendix 6). The costs and benefits are presented from Geneva University Hospitals’ perspective and could be extrapolated to the Swiss health care system. The cost per error prevented for epinephrine was US $ 534, and the cost per ADE prevented was US $ 4808. For epinephrine, the ROI becomes positive and profitable after the 16th injection, which is economically compelling given its frequent use in PICUs and PEDs globally. In 2019, Geneva University Hospitals administered epinephrine 40 times. This finding for epinephrine can be extended to other specific drugs. Similarly, considering the cost-effectiveness of midazolam, norepinephrine, and dopamine, it indicates that numerous other medications could derive advantages from the app for safe preparation and administration, especially high-risk drugs susceptible to significant patient harm if improperly prepared (eg, chemotherapeutics, narcotics, anticoagulants, electrolytes). Furthermore, with no upfront development costs and only maintenance expenses, if the app is purchased, achieving a positive ROI would be accelerated, and the additional cost of preventing an ADE would be reduced.

### Limitations and Future Work

This study has limitations, primarily related to the composite study framework used. First, ADE estimates were based on existing literature, which remains scarce in this field and limits generalization. Second, the partial reliance on data from simulated trials may be critiqued for its lack of external validity. However, high-fidelity simulations have been shown to effectively simulate real-life scenarios for evaluating research questions and technologies that are challenging to assess in real-world settings [43]. In addition, there is currently no data available on whether mobile apps reduce medication errors or ADEs in real-life settings. It is plausible that medication errors could potentially be higher in routine practice without app support due to external factors not accounted for in simulated trials. If so, this could underestimate the app’s ability to further reduce medication errors and ADEs in real-world settings and, consequently, the marginal cost of error reduction.

Third, while evidence suggests comparable medication error rates between in-hospital and prehospital settings [20,21], prehospital medication error rates were applied to the in-hospital setting, acknowledging differences in contexts and health care providers involved. Fourth, data specific to drug volumes used in the PED of Geneva University Hospitals were not available as they were not separately recorded. Therefore, the number of drug administrations required to achieve a positive ROI was estimated based on drug volumes administered in the PICU of Geneva University Hospitals, assuming app use in both PED and PICU settings.

Fifth, the reported error rate from the literature may have been underestimated, as medication errors are often underreported due to a lack of recognition or reluctance to report [44-46]. Sixth, our study’s cost list was not exhaustive. Extremely rare ADEs, such as death due to medication errors in pediatric care, were not included. In addition, indirect economic or health impacts, such as the effect of medication errors on patient quality of life, were not considered, presenting a conservative view of potential costs related to medication errors.

Finally, hospital systems may prefer integrated solutions within their workflows, and requiring staff to use personal devices without compensation could present challenges. However, PedAMINES, as an evidence-based app, provides a reliable and immediate solution to enhance medication safety, which may not always be achievable with existing hospital systems or in low-resource settings. Previous publications have demonstrated the app’s usability and technology acceptance during simulated pediatric in- and out-of-hospital CPR, affirming its practical applicability and effectiveness [47].

While this study primarily focused on the use of PedAMINES for epinephrine, we have already assessed the app’s effectiveness for other direct intravenous emergency drugs (midazolam, 10% dextrose, and sodium bicarbonate) [20], as well as for continuous infusions (dopamine and norepinephrine) [21]. The consistent decrease in risk to approximately 5% for all drugs, regardless of their varying degrees of preparation difficulty, reflects the app’s ability to secure the preparation stage of the medication process, irrespective of the drugs. Future work should include real-world studies to validate the app’s broader applicability and potential to reduce medication errors across different pediatric settings, such as onco-hematology or in low-resource countries. In addition, future studies should address the effectiveness of the app concerning age or weight categories separately. This approach will provide a clearer understanding of the factors influencing error rates in medication administration.

### Comparison With Previous Work

The literature has evaluated various interventions to reduce the economic burden of medication errors, including electronic systems, process interventions, patient-centered approaches, and interprofessional education programs [1]. Specifically, smart pumps have demonstrated cost-effectiveness in pediatric care by reducing administration errors [48,49], achieving a positive ROI of 1.15. At Geneva University Hospital (HUG) in 2019, using PedAMINES for 40 annual epinephrine administrations would have yielded an ROI of 2.5. Unlike the previous trials of the authors, the impact of a mobile app on reducing drug administration errors in pediatric care has not yet been extensively studied. The findings of this study underscore the potential of consumer-grade technology to enhance medical care efficiency in preparing and administering weight-based emergency drug doses, thereby improving medication safety.

PedAMINES has effectively reduced medication errors and ADEs during the administration of pediatric drug doses, particularly with epinephrine, at a reasonable cost per ADE prevented. In the Netherlands, Jeserun et al [50] reported a cost of €17.69 (~US $19.16) per error prevented using barcode administration, which is lower than our findings with PedAMINES. However, this discrepancy may be influenced by the specific focus of this study, which evaluated the app’s benefits with a limited range of drugs and settings.

These results are notably influenced by several key parameters: the cost of complications, the expenses for maintenance and usage, and the probability of ADEs. Given the constraints of our study and the limited evidence available for the Swiss context, we made necessary assumptions for these variables.

First, the annual maintenance and usage costs were estimated at 20% of the development cost, based on established models in the literature [23,24]. This figure reflects insights provided by PedAMINES developers regarding the ongoing resources required for app maintenance.

Second, the probability of ADE was informed by literature specific to pediatric emergency settings [28]. A conservative approach was adopted by assuming no fatalities associated with ADEs were prevented by the app, potentially underestimating its overall benefits. In England, drug error reduction software has been shown to prevent approximately 100 deaths across 745,170 admissions, with more harmful errors reported for pediatric patients [51]. While it is challenging to determine the exact extent to which medication errors in pediatric care lead to fatalities, these results suggest that the use of PedAMINES could provide nonaccounted benefits, potentially preventing deaths due to medication errors and thereby yielding significant cost savings.

Our MC simulations, accounting for parameter uncertainty, demonstrated that a positive ROI was achieved in 95% of scenarios after just 23 epinephrine administrations. This contrasts with the 40 annual epinephrine administrations at HUG, highlighting the potential for PedAMINES to yield positive economic outcomes when scaled nationally or internationally and applied to multiple drugs.

Further research, ideally on a national or multinational scale, would be essential to confirm the broader impact and cost-effectiveness of integrating PedAMINES across various health care settings.

### Conclusions

This study highlights PedAMINES as a consumer-grade, cost-effective mobile app designed to enhance medication safety, particularly in pediatric emergency care. This demonstrates its ability to swiftly achieve a positive ROI and underscore its potential economic value, especially if expanded to include other medications. However, the interpretation of the findings is constrained by the limitations inherent in literature review and simulation-based methodologies. Moving forward, it is crucial to validate in real-world settings whether the reduction in medication errors and ADEs achieved with PedAMINES translates into tangible economic benefits and improved patient outcomes. This empirical validation will provide deeper insights into the app’s practical impact and its potential role in enhancing pediatric health care delivery.

## Appendix

Multimedia Appendix 1 Average increase in cost weight in the cardiovascular category, 2019 (Swiss Diagnosis Related Groups).

Multimedia Appendix 2 Variables and distributions used in the analysis.

Multimedia Appendix 3 Sensitivity analysis with device cost added.

Multimedia Appendix 4 Sensitivity analysis, cumulative frequencies: norepinephrine, midazolam, and dopamine.

Multimedia Appendix 5 Deterministic sensitivity analysis (tornado plot): norepinephrine, midazolam, and dopamine.

Multimedia Appendix 6 Review of the economic evidence.

## Abbreviations

ADE: adverse drug event
CPR: cardiopulmonary resuscitation
HIT: health information technology
HUG: Geneva University Hospital
ICER: incremental cost-effectiveness ratio
LOS: length of stay
MC: Monte Carlo
PED: pediatric emergency department
PedAMINES: Pediatric Accurate Medication in Emergency Situations
PICU: pediatric intensive care unit
ROI: return on investment
