# Supplementary material for: Evaluating the Economic Impact of the PedAMINES App in Reducing Medication Errors in Pediatric Emergency Care: Cost-Effectiveness Analysis
Source: J Med Internet Res. 2024 Oct 25;26:e52077. doi: 10.2196/52077 (PMC11549577; doi:10.2196/52077)
Supplement: Multimedia Appendix 1 [file jmir_v26i1e52077_app1.docx]

**Multimedia Appendix 1.** Average increase in cost weight in the cardiovascular category, 2019 (Swiss Diagnosis Related Groups).

| DRG | Name | no complications | complication | increase |
| --- | --- | --- | --- | --- |
|  |  |  |  |  |
| F60A-F60B | Acute myocardial infarction without invasive cardiological diagnostic procedures | 0.692 | 1.477 | 113.44 |
| F70A-F70B | Severe arrhythmia and cardiac arrest | 0.698 | 1.47 | 110.60 |
| F59B-F59G | complex or moderately complex vascular procedures | 0.785 | 2.685 | 242.04 |
| F62A-F62C | Heart failure and shock | 0.888 | 1.846 | 107.88 |
| F01D-F01E | Placement of a cardioverter defibrillator, dual-chamber stimulation | 4.563 | 5.833 | 27.83 |
| F03B-F03C | Heart valve operation with heart-lung machine, with complication procedure or pacemaker, or triple operation or age < 1 year or under deep hypothermia or with bio prosthesis and specific heart valve replacement | 4.846 | 6.568 | 35.53 |
| F60D-F06C | Coronary bypass surgery | 3.093 | 3.915 | 26.58 |
| F12E-F12B | Implantation of a pacemaker with single-chamber pacing | 1.605 | 3.255 | 102.80 |
| F18A-F18B | Revision of a cardiac pacemaker or cardioverter defibrillator without changing aggregates | 1.264 | 3.124 | 147.15 |
| F21C-F21B | Other surgical procedures for cardiovascular diseases | 1.406 | 1.524 | 8.39 |
| F31C-F31A | Other cardiovascular procedures with heart-lung machine, age > 0 years | 3.259 | 5.316 | 63.12 |
| F34D-F34C | Other major vascular reconstructive procedures without heart-lung machine | 1.94 | 3.495 | 80.15 |
| F39C-F39A | Vein ligation and stripping | 0.623 | 0.8 | 28.41 |
| F41B-F41A | Invasive cardiological diagnostic procedures for acute myocardial infarction | 0.964 | 1.932 | 100.41 |
| F49D-F49B | Invasive cardiological diagnostic procedures except for acute myocardial infarction, age > 13 years | 0.883 | 1.539 | 74.29 |
| F63B-F63A | Venous thrombosis | 0.568 | 0.929 | 63.56 |
| F67B-F67A | Hypertension | 0.476 | 0.984 | 106.72 |
| F69B-F69A | Valvulopathy | 0.569 | 1.788 | 214.24 |
|  |  |  |  |  |
|  |  |  | Mean | 91.84 |
