# Supplementary material for: Evaluating the Economic Impact of the PedAMINES App in Reducing Medication Errors in Pediatric Emergency Care: Cost-Effectiveness Analysis
Source: J Med Internet Res. 2024 Oct 25;26:e52077. doi: 10.2196/52077 (PMC11549577; doi:10.2196/52077)
Supplement: Multimedia Appendix 2 [file jmir_v26i1e52077_app2.docx]

**Multimedia Appendix 2.** Variables and distributions used in the analysis^a,b^

| Parameter | Distribution | Mean | SD | CI 95% | Ref. |
| --- | --- | --- | --- | --- | --- |
|  |  |  |  |  |  |
| Total number of dopamine administrations investigated in 2019 hospital experiment | Fixed Value | 64 |  | - | [1] |
| Total number of norepinephrine administrations investigated in 2019 hospital experiment | Fixed Value | 64 |  | - | [1] |
| Total number of epinephrine administrations investigated in 2021 pre-hospital experiment | Fixed value | 76 |  | - | [2] |
| Total number of midazolam administrations investigated in 2021 pre-hospital experiment | Fixed value | 76 |  | - | [2] |
| Preparation time to intravenous administration of dopamine/norepinephrine in the experimental group (i.e., with app support), in seconds | Normal | 181.4 | 54.7 | ]74.2 ; 288.6[ | [1] |
| Preparation time to intravenous administration of dopamine/norepinephrine in the control group (i.e., without app support), in seconds | Normal | 329.60 | 143.1 | ]49.1 ; 610.1[ | [1] |
| Preparation time to intravenous administration of midazolam in the experimental group (i.e., with app support), in seconds | Normal | 145.4 | 47.6 | ]52.1 ; 238.7[ | [2] |
| Preparation time to intravenous administration of midazolam in the control group (i.e., without app support), in seconds | Normal | 173.5 | 103.1 | ]0 ; 375.6[ | [2] |
| Preparation time to intravenous administration of epinephrine in the experimental group (i.e., with app support), in seconds | Normal | 191.6 | 80.3 | ]34.2 ; 349.0[ | [2] |
| Preparation time to intravenous administration of epinephrine in the control group (i.e., without app support), in seconds | Normal | 200.1 | 73.4 | ]56.2;344.0[ | [2] |
| Number of dopamine overdoses (>10% prescribed dose) in the control group | Uniform | 16.0 | - | ]12.8 ; 19.2[ | [1] |
| Number of dopamine overdoses (>10% prescribed dose) in the experimental group | Uniform | 2.0 | - | ]1.6 ;2.4[ | [1] |
| Number of norepinephrine overdoses (>10% prescribed dose) in the control group | Uniform | 3.0 | - | ]2.4 ;3.6[ | [1] |
| Number of norepinephrine overdoses (>10% prescribed dose) in the experimental group | Uniform | 1.0 | - | ]0.8 ;1.2[ | [1] |
| Number of dopamine underdoses (>10% prescribed dose) in the control group | Uniform | 22.0 | - | ]17.6 ;26.4[ | [1] |
| Number of dopamine underdoses (>10% prescribed dose) in the experimental group | Uniform | 4.0 | - | ]3.2 ;4.8[ | [1] |
| Number of norepinephrine underdoses (>10% prescribed dose) in the control group | Uniform | 31.0 | - | ]24.8 ;37.2[ | [1] |
| Number of norepinephrine underdoses (>10% prescribed dose) in the experimental group | Uniform | 2.0 | - | ]1.6 ;2.4[ | [1] |
| Number of midazolam overdoses (>10% prescribed dose) in the control group | Uniform | 28.0 | - | ]22.4 ;33.6[ | [2] |
| Number of midazolam overdoses (>10% prescribed dose) in the experimental group | Uniform | 5.0 | - | ]4.0 ; 6.0[ | [2] |
| Number of epinephrine overdoses (>10% prescribed dose) in the control group | Uniform | 7 | - | ]5.6;8.4[ | [2] |
| Number of epinephrine overdoses (>10% prescribed dose) in the experimental group | Uniform | 2.0 | - | ]1.6 ; 2.4[ | [2] |
| Number of midazolam underdoses (>10% prescribed dose) in the control group | Uniform | 28.0 | - | ]22.4 ;33.6[ | [2] |
| Number of midazolam underdoses (>10% prescribed dose) in the experimental group | Uniform | 0.0 | - | ]0 ;0[ | [2] |
| Number of epinephrine underdoses (>10% prescribed dose) in the control group | Uniform | 36.0 | - | ]28.8 ;43.2[ | [2] |
| Number of epinephrine underdoses (>10% prescribed dose) in the experimental group | Uniform | 2.0 | - | ]1.6 ; 2.4[ | [2] |
| Probability of an ADE, used for dopamine | Normal | 11.1% | 1.11% | ]9.1% ;13.5%[ | [3] |
| Probability of an ADE, used for norepinephrine | Normal | 11.1% (1.11%) ]9.1% ;13.5%[ | 1.11% | ]9.1% ;13.5%[ | [3] |
| Probability of an ADE, used for midazolam | Normal | 11.1% (1.11%) ]9.1% ;13.5%[ | 1.11% | ]9.1% ;13.5%[ | [3] |
| Probability of an ADE, used for epinephrine | Normal | 11.1% (1.11%) ]9.1% ;13.5%[ | 1.11% | ]9.1% ;13.5%[ | [3] |
| Probability of temporary harm to the patient and need for initial or prolonged hospitalization (category F) | Normal | 3% | 1.89% | ]0.6% ; 8.0%[ | [3] |
| Probability of temporary harm to the patient and complication (category E) | Normal | 97% | 1.89% | ]92.0% ;99.4%[ | [3] |
| Daily cost of an in intensive care unit inpatient stay, in USD, 2019 | Uniform | 229.7 | - | ]183.8 ; 275.7[ | Geneva University Hospitals accounting |
| Increased cost of hospitalization due to complication: 91.84% of ICU cost, in USD, 2019 | Uniform | 22,080.0 | - | ]17664.0; 267496.0[ | (Appendix 4) |
| Costs of using and maintaining the app, including the software and resources devoted to using the app each year. | Uniform | 37,205.3 | - | ]29764.2 ; 44646.3[ | Expert opinion + [4, 5] |
| Cost of training any nurse in the PED, in USD, 2019 | Uniform | 295.2 | - | ]236.1; 354.2[ | Geneva University Hospitals accounting + expert opinion |
| Cost of training any physician in the PED, in USD, 2019 | Uniform | 174.2 | - | ]139.4; 209.1[ | Geneva University Hospitals accounting + expert opinion |
| Conditional length of stay; i.e., number of days of prolonged hospitalization in the event of an ADE. | Normal | 0.6 | 0.1 | ]0.4 ; 0.9[ | [6] |
| Exchange rate USD :CHF as at December 31, 2019 | Fixed value | 1.03 | - | - | [7] |
| The number of Dopamine administration in PICU, 2019 | Not used in sensitivity analysis | 100 | - | - | Geneva University Hospitals accounting |
| The number of epinephrine administrations PICU, 2019 | Not used in sensitivity analysis | 40 | - | - | Geneva University Hospitals accounting |
| The number of midazolam administrations in PICU, 2019 | Not used in sensitivity analysis | 250 | - | - | Geneva University Hospitals accounting |
| The number of norepinephrine administrations in PICU, 2019 | Not used in sensitivity analysis | 141 | - | - | Geneva University Hospitals accounting |

^a^Table of variables and distributions used in the analysis. Where the information on the distribution of the variable was not available, A uniform distribution with a confidence interval of +- 20% was assumed.

^b^SD: Standard Deviation of the normal distribution, CI: confidence interval, ADE: adverse drug event, PICU: pediatric intensive care unit, PED: pediatric emergency department

Reference list :

1. Siebert JN, Ehrler F, Combescure C, Lovis C, Haddad K, Hugon F, et al. A mobile device application to reduce medication errors and time to drug delivery during simulated paediatric cardiopulmonary resuscitation: a multicentre, randomised, controlled, crossover trial. The Lancet Child & Adolescent Health. 2019;3(5):303-11. doi: 10.1016/S2352-4642(19)30003-3

2. Siebert JN, Bloudeau L, Combescure C, Haddad K, Hugon F, Suppan L, et al. Effect of a mobile app on prehospital medication errors during simulated pediatric resuscitation: a randomized clinical trial. JAMA network open. 2021;4(8):e2123007-e. doi: 10.1001/jamanetworkopen.2021.23007

3. Takata GS, Mason W, Taketomo C, Logsdon T, Sharek PJ. Development, testing, and findings of a pediatric-focused trigger tool to identify medication-related harm in US children's hospitals. Pediatrics. 2008;121(4):e927-e35. doi: 10.1542/peds.2007-1779

4. Ren Y, Xing T, Chen X, Chai X, editors. Research on software maintenance cost of influence factor analysis and estimation method. 2011 3rd International Workshop on Intelligent Systems and Applications; 2011: IEEE. doi: 10.1109/ISA.2011.5873461

5. Singh C, Sharma N, Kumar N. Analysis of software maintenance cost affecting factors and estimation models. Int J Sci Technol Res. 2019;8:276-81. doi:

6. Oderda GM, Said Q, Evans RS, Stoddard GJ, Lloyd J, Jackson K, et al. Opioid-related adverse drug events in surgical hospitalizations: impact on costs and length of stay. Annals of Pharmacotherapy. 2007;41(3):400-7. doi: 10.1345/aph.1H386

7. IMF. Representative Exchange Rates for Selected Currencies for December 201. International Monetary Found. 2019. doi: https://www.imf.org/external/np/fin/data/rms_mth.aspx?SelectDate=2019-12-31&reportType=REP (visited on January 17, 2023)
