# Supplementary material for: Evaluating the Economic Impact of the PedAMINES App in Reducing Medication Errors in Pediatric Emergency Care: Cost-Effectiveness Analysis
Source: J Med Internet Res. 2024 Oct 25;26:e52077. doi: 10.2196/52077 (PMC11549577; doi:10.2196/52077)
Supplement: Multimedia Appendix 3 [file jmir_v26i1e52077_app3.docx]

**Multimedia Appendix 3.** Sensitivity analysis with device cost added.

| Drugs | Number of preventable errors per administration | Number of preventable ADE per administration | Cost per prevented error, in USD | Cost per prevented ADE, in USD |
| --- | --- | --- | --- | --- |
|  |  |  |  |  |
| Epinephrine (direct IV) | 0.513 | 0.057 | 542 | 4’883 |
| Dopamine (continuous infusion) | 0.500 | 0.056 | 784 | 7’065 |
| Norepinephrine (continuous infusion) | 0.484 | 0.054 | 1094 | 9’856 |
| Midazolam (direct IV) | 0.671 | 0.074 | 234 | 2’106 |
