# Supplementary material for: Evaluating the Economic Impact of the PedAMINES App in Reducing Medication Errors in Pediatric Emergency Care: Cost-Effectiveness Analysis
Source: J Med Internet Res. 2024 Oct 25;26:e52077. doi: 10.2196/52077 (PMC11549577; doi:10.2196/52077)
Supplement: Multimedia Appendix 4 [file jmir_v26i1e52077_app4.docx]

**Multimedia Appendix 4.** Sensitivity analysis, cumulative frequencies: norepinephrine, midazolam, and dopamine^a^.

^a^Cumulative frequency of number of administrations required to achieve a positive ROI, on 10,000 MC simulations. The grey line represents the threshold below which 95% of the simulations fall. The black line represents the cumulative frequency of the MC simulations.
