# Supplementary material for: Evaluating the Economic Impact of the PedAMINES App in Reducing Medication Errors in Pediatric Emergency Care: Cost-Effectiveness Analysis
Source: J Med Internet Res. 2024 Oct 25;26:e52077. doi: 10.2196/52077 (PMC11549577; doi:10.2196/52077)
Supplement: Multimedia Appendix 5 [file jmir_v26i1e52077_app5.docx]

**Multimedia Appendix 5.** Deterministic sensitivity analysis (tornado plot): norepinephrine, midazolam, and dopamine^a,b^

^a^Relative importance of the uncertainty according to the variables used in the analysis.

^b^prob: probability, cat: category, nor: norepinephrine, mid: midazolam, dop: dopamine, OD: Overdoses, UD: Underdoses, exp group: experimental group, ctrl group: control group, CLOS: conditional length of stay,
