# Supplementary material for: Evaluating the Economic Impact of the PedAMINES App in Reducing Medication Errors in Pediatric Emergency Care: Cost-Effectiveness Analysis
Source: J Med Internet Res. 2024 Oct 25;26:e52077. doi: 10.2196/52077 (PMC11549577; doi:10.2196/52077)
Supplement: Multimedia Appendix 6 [file jmir_v26i1e52077_app6.docx]

**Multimedia Appendix 6.** Review of the economic evidence.

To obtain an overview of modelling approaches and extract relevant economic evidence in this area, we first conducted a systematic review of recently published studies that aimed to measure the costs of medication errors in pediatric care and the cost-effectiveness of interventions to reduce them. We searched the major medical databases [PubMed (National Center for Biotechnology Information) and EMBASE (Excerpta Medica dataBASE)] and reviewed the English-language biomedical literature. All available articles related to medication errors and economic evaluation in pediatric care were retrieved and reviewed. The search period was arbitrarily extended from 1 January 2010 to 31 January 2021. The following Medical Subject Headings (MeSH) and keywords from the study domains were searched and linked using Boolean operators: ('medication error'/exp OR 'inappropriate prescribing'/exp OR 'drug administration error*':ti,ab OR 'drug errors':ti,ab OR 'inappropriate medication':ti,ab OR 'inappropriate prescri*':ti,ab OR 'medication administration error*':ti,ab OR 'medication discrep*':ti,ab OR 'medication error*':ti,ab OR 'over prescri*':ti,ab OR 'preventable adverse drug event*':ti,ab OR 'preventable ADE':ti,ab OR 'preventable adverse drug reaction*':ti,ab OR 'wrong prescri*':ti,ab OR ((risk* NEAR/3 medication*):ti,ab)) AND ('cost'/exp OR 'pharmacoeconomics'/de OR cost*:ti,ab OR econ*:ti,ab OR pharmacoecon*:ti,ab) AND ('intensive care unit'/exp OR 'hospital emergency service'/de OR emergenc*:ti,ab OR icu:ti,ab OR inpatient*:ti,ab OR 'intensive care unit*':ti,ab OR picu:ti,ab OR 'pre-hospital care':ti,ab OR ward*:ti,ab) AND ('child'/exp OR babies:ti,ab OR baby:ti,ab OR boy:ti,ab OR boys:ti,ab OR child*:ti,ab OR girl*:ti,ab OR infant*:ti,ab OR neonatal:ti,ab OR paediatric*:ti,ab OR pediatric*:ti,ab). All references provided in the original manuscript were retrieved for other relevant citations and the related articles retrieved. This process was repeated until the reference lists covered the full set of articles already retrieved. All articles whose titles or abstracts included the search terms and costs were screened and those corresponding to the research focus were included in the review. We specifically focused on drug medication process steps combined with IT procedures and costs, ICER or ROI where available.

Table S1. Table of the 9 studies extracted from the biomedical literature review^a^

| Articles | Year | Country | Study design | Stage of the medication management process studied (prescription, transcription, preparation, administration, monitoring) | Economic findings |
| --- | --- | --- | --- | --- | --- |
|  |  |  |  |  |  |
| [1] | 2020 | UK | experimental study and questionnaire | administration | Cost savings over 1 year: £20022 for the hospital, £4819 for the NHSE |
| [2] | 2020 | Canada | observational study | prescription, transcription, preparation, administration | AE patients had higher costs than non-AE patients |
| [3] | 2019 | Brazil | MC simulation based on observational data | preparation, administration | Drug library infusion pumps are more effective than traditional approaches |
| [4] | 2019 | Italy | experimental controlled study (in vitro) | preparation | Robotic therapy reduced the costs especially when multiple preparations were required |
| [5] | 20182018 | USA | experimental study | administration | Actual charge savings due to reduction errors: $165,000 to $255,000 |
| [6] | 2014 | Spain | interventional prospective study | administration | The implementation of smart pump technology saved costs associated with preventable ADEs (the estimated cost of an AE would have to be euros 3093.5). |
| [7] | 2011 | England | retrospective analysis | not specified | Total cost of litigation ranged from £600 to £3044943 |
| [8] | 2010 | Switzerland | qualitative and quantitative risk assessment | prescription, transcription, preparation, administration | The best cost-effectiveness ratio was 1 quali = euros 0.54 for "clinical pharmacist" |
| [9] | 2021 | Japan | observational | not specified | National cost of ADE: from USD 141 to 588 million |

^a^MC: Monte-Carlo, AE: Adverse Events, NHSE: National Health Service of England

reference list:

1. Balfour-Lynn IM, Khan K, Pentayya N, Pheasant C, Bentley S, Makhecha S. Initiating Self-Administration of Medicines for inpatients with cystic fibrosis. Archives of Disease in Childhood. 2020;105(10):986-90. doi:

2. Stewart TC, Luong K, Alharfi I, McKelvie B, Fraser DD. Identification of adverse events in pediatric severe traumatic brain injury patients to target evidence-based prevention for increased performance improvement and patient safety. Injury. 2020;51(7):1568-75. doi:

3. Silva RCLd, QuinellatoLouro T, Peregrino AAdF, Silva CRLd, Marta CB, Itria A. Cost-effectiveness of infusion pumps to reduce errors in a Pediatric ICU. Revista Brasileira de Enfermagem. 2019;72:617-23. doi: 10.1590/0034-7167-2018-0526

4. Amodeo I, Pesenti N, Raffaeli G, Sorrentino G, Zorz A, Traina S, et al. Robotic therapy: Cost, accuracy, and times. New challenges in the neonatal intensive care unit. Frontiers in Pharmacology. 2019;10:1431. doi:

5. Hebbar KB, Colman N, Williams L, Pina J, Davis L, Bost JE, et al. A quality initiative: a system-wide reduction in serious medication events through targeted simulation training. Simulation in Healthcare. 2018;13(5):324-30. doi:

6. Manrique-Rodríguez S, Sánchez-Galindo AC, López-Herce J, Calleja-Hernández MÁ, Martínez-Martínez F, Iglesias-Peinado I, et al. Implementing smart pump technology in a pediatric intensive care unit: a cost-effective approach. International Journal of Medical Informatics. 2014;83(2):99-105. doi: 10.1016/j.ijmedinf.2013.10.011

7. Raine JE. An analysis of successful litigation claims in children in England. Archives of disease in childhood. 2011;96(9):838-40. doi:

8. De Giorgi I, Fonzo-Christe C, Cingria L, Caredda B, Meyer V, Pfister RE, et al. Risk and pharmacoeconomic analyses of the injectable medication process in the paediatric and neonatal intensive care units. International journal for quality in health care. 2010;22(3):170-8. doi:

9. Iwasaki H, Sakuma M, Ida H, Morimoto T. The burden of preventable adverse drug events on hospital stay and healthcare costs in Japanese pediatric inpatients: The JADE Study. Clinical Medicine Insights: Pediatrics. 2021;15:1179556521995833. doi: 10.1177/117955652199583
